# Exploring the dynamics of exercise intensity on male fertility and reproductive health: advancements and implications for fertility research

**DOI:** 10.3389/frph.2024.1423916

**Published:** 2024-07-18

**Authors:** Navid Abedpoor, Farzaneh Taghian, Fatemeh Hajibabaie

**Affiliations:** ^1^ Department of Sports Physiology, Faculty of Sports Sciences, Isfahan (Khorasgan) Branch, Islamic Azad University, Isfahan, Iran; ^2^ Department of Physiology, Medicinal Plants Research Center, Isfahan (Khorasgan) Branch, Islamic Azad University, Isfahan, Iran; ^3^ Department of Biology, Faculty of Basic Sciences, Shahrekord Branch, Islamic Azad University, Shahrekord, Iran

**Keywords:** infertility, oxidative stress, exercise, physical activity, inflammation

## Abstract

On a global scale, 15% of couples experience infertility. Approximately 50% of these cases refer to male infertility. This literature review investigated the effects of exercise activities on male fertility and reproductive health. This subject was explored using studies conducted on human and animal models. Physical activity is widely recognized to improve overall well-being, but engaging in excessive exercise might potentially lead to male infertility due to the negative impact on hypothalamic-pituitary-testicular (HPT) function, increased oxidative stress, and the presence of chronic inflammation. Infertility might result from the fundamental factors that induce a decline in testosterone production and semen quality. Physical activity has the potential strategy to enhance male fertility in cases of infertility caused by obesity and diabetes. Exercise enhances spermatogenesis and semen quality in lifestyle-induced infertility by increasing testicular antioxidant defense, reducing pro-inflammatory cytokines, and promoting steroidogenesis.

## Introduction

Although the influence of lifestyle behaviors such as cigarette smoking, poor nutrition, excessive drinking, overweight and obesity, psychological stress, and lack of physical activity on male reproduction is well-established, the effects of physical activity and exercise training on reproductive performance remain primarily unnoticed. Several studies have revealed that engaging in extended and intense exercise may negatively impact various physiological systems, specifically the reproductive system and fertility (1, 2). However, some proponents argue that regular exercise positively influences an individual’s overall health and well-being. Over the last decade, researchers have highlighted the harmful impact of exercise on male reproductive processes (3).

Studies have shown that intense endurance exercise may harm reproductive hormones and semen quality (1, 3, 4). Moreover, growing evidence has shown that vigorous exercise may lead to oxidative stress and DNA damage in the sperm of male athletes (5, 6). However, further research indicated that the effects of exercise intensity on male fertility were at least as significant as the adverse effects caused by exercise volume.

It is essential to consider that physical activity, exercise, and sports training have distinct meanings, demands, progression, and goals (5). Physical activity encompasses any movement that necessitates the contraction of muscles. Physical activity includes routine activities, such as horticulture, ambulation, ascending and descending stairs, domestic chores, and any other tasks performed for the day (5). In contrast, exercise is a deliberate and organized action to obtain health advantages and improve physical fitness (7). Endurance training primarily focuses on developing the aerobic rather than the anaerobic system. It encompasses several systemic processes and events, leading to central and peripheral physiological adaptations (8). Furthermore, catabolic and oxidation processes play a vital role in enhancing the ability to use fat and glycogen to fulfill energy requirements (glycogenolysis, glycolysis, and lipolysis), as well as improving the efficiency of oxygen transportation and distribution (8).

The research found a strong positive connection between sperm DNA fragmentation and VO_2max_, seminal 8-isoprostane, ROS, and malondialdehyde (MDA) levels. These results indicate that extensive exercise training is at a higher risk of experiencing sperm malfunction compared to males who engage in leisure exercise or have sedentary lifestyles (6). Vaamonde and coworkers have revealed a direct correlation between cycle volume and sperm DNA damage. Thus, athletes that exhibited the most significant sperm DNA damage consistently engaged in greater weekly training volume on average each year (7). A study was conducted to examine the impact of exercise on reproductive hormones and semen quality. The study included 286 participants who were divided into two groups; one group performed moderate-intensity exercise at 60% of their maximum oxygen consumption (VO_2max_), while the other group performed high-intensity exercise at 80% of their VO_2max_ (1). In this study, FSH, LH, PRL, DHEA, and cortisol levels were changed in individuals who conducted high-intensity exercise for two weeks. However, no significant alterations were found in testosterone, progesterone, or estradiol levels. After three days of recuperation, hormone levels were restored to their original values before the training period (7).

Based on the mentioned evidence, this review discusses the relationship between exercise training intensity and the molecular mechanisms involved in reactive oxygen species (ROS), which may impact sperm quality.

### Relationship between different levels of physical activity and reproductive health outcomes

The endurance exercise and high-intensity interval training (HIIT) protocols effectively enhanced glucose metabolism and reduced central fat buildup in the trained groups (9, 10). The effectiveness of endurance exercise and HIIT programs ameliorate metabolic alterations without negatively affecting reproductive parameters (9). Research conducted on both humans and animals indicated that making lifestyle modifications, such as engaging in regular exercise and following a restricted diet, may lead to enhanced semen quality in those who are obese (11, 12). Ibáñez et al. assessed the impact of moderate-intensity aerobic training on the reproductive parameters of rats fed a high-fat diet during adolescence (13). The training protocol successfully reversed the adverse changes in these animals’ reproductive parameters within four weeks (55%–65% of VO_2max_ for 20 min) (13).

Nevertheless, the connection between exercise duration, intensity, and impact on reproductive ability is still ambiguous. According to a review by Hayden et al., men who engage in high volumes and intensities of training may experience changes in various reproductive parameters (14). These changes include alterations in sperm morphology, concentration, and motility, as well as reduced levels of luteinizing hormone, follicle-stimulating hormone, and testosterone (14). Prolonged aerobic training programs of moderate intensity enhance the skeletal muscle’s oxidative capacity (14). The moderate intensity might modify the energy source used during exercise and ultimately improve aerobic capacity. Recent studies have shown that high-intensity interval training (HIIT) changes skeletal muscle utilization energy and has similar effects to moderate-intensity aerobic exercise (15). Vaamonde et al. showed that higher training intensity had detrimental impacts on seminiferous characteristics, particularly concerning sperm morphology (16). Wise et al. discovered that a weekly volume of more than 5 h was linked to reduced sperm concentrations (17). Safarinejad et al. reported adverse impacts on sperm quality in males who underwent high-intensity or high-volume regimens (1). These data suggest that insufficient quantification of these two factors might severely impact male fertility.

Performing aerobic exercises has the potential to mitigate oxidative damage in testicular tissue, a condition that is exacerbated by metabolic syndrome. During anaerobic activities, it was observed that the same phenomenon occurred in the opposite direction due to the increasing intensity of the activity (1, 18). HIIT is inherently a physiological stressor for the body. While acknowledging the benefits of high-intensity loads, it is essential to consider that the occurrence of oxidative stress might have detrimental consequences on the reproductive system (18). Engaging in moderate-intensity exercise could help mitigate the adverse impact of obesity on male reproductive function. Our hypothesis suggests a potential correlation between this occurrence and oxidative stress and the inflammatory response (8).

Male obesity disturbs the equilibrium between oxidation and antioxidation in the testicular tissue, leading to oxidative stress (19). This stress activates NF-κB and initiates an inflammatory response, decreasing testosterone production and impairing sperm quality (20). Moderate-intensity physical activity mitigated the elevated oxidative stress caused by obesity, suppressed the activity of NF-κB and pro-inflammatory cytokines, and enhanced testosterone production and sperm quality (21). Nevertheless, engaging in high-intensity exercise did not mitigate oxidative stress and inflammatory response caused by obesity in the testicular tissue, nor did it enhance the diminished male reproductive function (22). Thus, the oxidative stress-inflammatory response induced by intense exercise may have counteracted the inhibitory effects of reducing body fat on oxidative stress. Thus, it is hypothesized that various exercise routines have varying impacts on male reproductive function affected by obesity via the suppression/activation of oxidative stress inflammatory response (23).

Cyclists have been found to have decreased sperm motility (46.2 ± 19.5%) during competitive times comparison to other groups, such as recreational marathon participants and sedentary participants amid their concurrence periods (*P* < 0.05), as well as compared to themselves during the other two times of study (*P* < 0.01) (24, 25). Further research revealed that long-distance competitive cyclists had a significantly lower percentage of spermatozoa with typical characteristics and a significantly more significant percentage of morphologically atypical pointed forms compared to control subjects (no significant difference was noticed in semen volume and sperm motility, viability, and concentration) (25). Even recreational athletes who vary their training and exercise to the point of fatigue exhibited changes in their semen and hormone levels (3, 26). In this study, we indicated the type, duration, and intensity of exercises on fertility and sperm quality in Table 1.

**Table 1:** Effect of exercise type, duration, and intensity on fertility and sperm quality.

Protocol	Plan			Type
	Duration	Intensity	Time	
Low-Moderate intensity	4 weeks	55%–65% of VO2max	20 min	Treadmill (13)
Moderate-Intensity	8 weeks	14–17.5 m/min	45 min	Treadmill (27)
	8 weeks	65%–70% VO2 max	60 min	Treadmill (28)
	6 weeks	-	120 min once per day	Free swimming (8)
	6 weeks	50%–60% of VO2max	20 min	Treadmill (18)
High-Intensity	8 weeks	24–27.5 m/min	1 min	Treadmill (27)
	8 weeks	85%–90% VO2 max	30 min	Treadmill (28)
	6 weeks	-	120 min twice per day	Free swimming (8)
	12 weeks	70%–85% of VO2max	40- to 50 min	Treadmill (29)

### Reactive oxygen species (ROS) and inflammation influence reproductive processes

Research has demonstrated that the demand for oxygen increases dramatically during physical activity, with the amount of oxygen consumed by muscles rising more than 100-fold compared to resting conditions. Furthermore, exercise increases the levels of free radicals within the body (30). Conversely, the rise in free radicals might trigger heightened antioxidant enzyme activity, safeguarding cells from harm caused by excessive free radical generation (31). The positive adaptation response often results in a substantial elevation in the blood testosterone level and improvements in the quality, count, and DNA integrity of the sperm in males (32). Excessive exercise might generate a significant quantity of free radicals that surpass the body’s ability to counteract them with antioxidants. This surplus of free radicals has the potential to harm male reproductive function.

Research has demonstrated that male rats who undergo intense physical activity had higher levels of oxidative stress capacity in testicular tissues, decreased antioxidant enzyme activities, reduced levels of key steroidogenic enzymes, testosterone synthesis, and spermatogenesis. These findings suggest a connection between the oxidative stress caused by strenuous exercise and reproductive dysfunction (33). Reactive oxygen species (ROS) are also crucial in sperm capacitation, although high amounts of ROS could cause oxidative damage. An imbalance in ROS levels and the mechanisms that regulate sperm quality might result from non-communicable disease or environmental variables. This imbalance could elevate oxidative stress, cellular damage, and apoptosis, decreasing sperm concentration, quality, and motility (8, 18). Thus, evaluating mitochondrial functioning and quality control is crucial to getting significant insights into male infertility.

**Figure 1:**
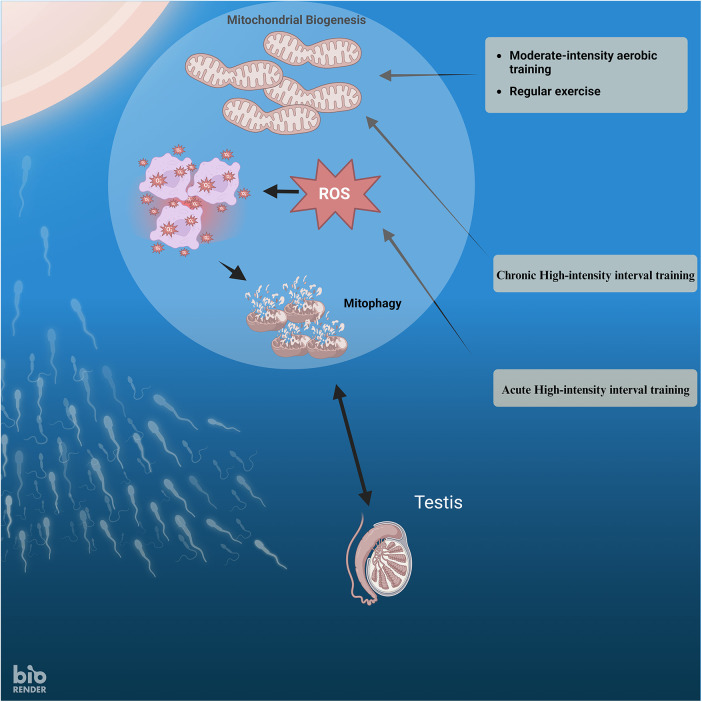
The relationship between exercise intensity, ROS, mitophagy, and mitochondrial biogenesis in sperm.

Maintaining optimal mitochondrial functioning is crucial for general well-being, specifically emphasizing male fertility. Evaluating the performance and maintenance of mitochondria may provide vital insights into investigating and treating male infertility, perhaps leading to the creation of novel management approaches (Figure 1). Within testicular tissue, mitochondria serve several roles, such as generating energy (34), producing steroid hormones in the testis (35), supporting cell proliferation, and facilitating cell death (36). Additionally, mitochondria-produced ROS are crucial in the physiological processes that allow sperm cells to fertilize an egg cell. These processes include biochemical alterations associated with tyrosine phosphorylation, liberation of cholesterol, and the contact between sperm and egg.

Each mitochondrion in human spermatozoa has just one copy of mtDNA, and the mtDNA sequence in spermatozoa is similar to that seen in somatic cells (37). The mitochondria in spermatozoa produce ATP necessary to facilitate sperm motility (38). Therefore, the proper functioning of the OXPHOS is required for the motility of human spermatozoa (38).

Numerous research have discovered an adverse correlation between the amounts of cytokines in the fluid that carries semen and standard indicators of sperm quality, as well as the integrity of sperm DNA (39, 40). Prior research has shown oxidative stress as a critical factor in either disrupting the function of sperm cells or causing damage to sperm DNA (41, 42). Elevated levels of DNA fragmentation in sperm cells are a crucial element that may have significant implications for pregnancy and subsequent embryonic development. Thus, it is feasible that using defensive methods to counteract oxidative stress and inflammation might serve as an effective therapeutic option for addressing male infertility (42).

Recent research has shown that regular exercise and physical activity positively affect inflammatory mediators and the redox state of cells and tissues (43). Studies have shown that persistent and vigorous exercise may impact indicators of male reproductive function in healthy individuals (43). Hajizadeh Maleki et al. conducted a study to assess the levels of pro-inflammatory cytokines, peroxidative and antioxidative biomarkers, semen quality, sperm DNA integrity, and pregnancy rate in sedentary, infertile individuals after 24 weeks of high-intensity exercise (43). After 24 weeks of the intervention, the average levels of ROS and malondialdehyde were considerably reduced compared to the initial values in the exercise (EX) group (*P* < 0.05). After exercising, the values reverted to their original baseline level during 30 days. After 24 weeks, the exercise group showed a substantial increase in superoxide dismutase, catalase, and total antioxidant capacity compared to the initial measurements (*P* < 0.05). Even after 30 days of detraining, the levels of SOD and catalase enzymes remained considerably higher (*P* < 0.05). However, the levels of TAC recovered to their original baseline values 30 days after the exercise. After 24 weeks of the intervention, the average levels of IL-6 and TNF-α were considerably reduced compared to the initial values in the exercise group (*P* < 0.05) (42, 43). Hence, exercise changes in seminal markers related to inflammation and oxidative stress.

Researchers have suggested that pro-inflammatory cytokines, such as IL-6 and TNF-α, in seminal plasma are linked to disruptions in semen parameters and the integrity of sperm DNA (44). Several studies have shown that the pro-inflammatory cytokines generated during inflammation or infection in semen also disrupt the balance between oxidants and antioxidants in the seminal plasma, resulting in peroxidative damage to spermatozoa and reduced fertility (45–47). Additionally, it has been proposed that exercise training reduces the activity of pro-inflammatory substances while increasing the production of anti-inflammatory substances in various bodily fluids, organs, and tissues (48, 49). These results contradict prior research showing an elevation in pro-inflammatory cytokines after 8 and 16 weeks of moderate to rigorous cycling training in male road bikers (50, 51). The variations in training methodologies used to assess chronic cytokine responses to exercise training and the diverse populations investigated might account for these discrepancies. The precise processes behind the improvements in semen characteristics and sperm DNA integrity produced by exercise are not well-defined. However, it seems that the decreases in pro-inflammatory cytokines in semen and the enhancement of the redox state mediate these benefits (52).

It is crucial to emphasize the significance of ROS in spermatozoa capacitation since ROS generation regulates protein tyrosine phosphorylation. ROS presence initiates a series of metabolic processes that enhance sperm motility. Initially, ROS stimulates the action of the enzyme adenylyl cyclase by transforming adenosine triphosphate (ATP) into cyclic adenosine monophosphate (cAMP). Subsequently, cAMP initiates the activation of protein kinase A (PKA), which in turn enhances the generation of ROS and the enzyme NADPH oxidase. PKA phosphorylates serine and tyrosine residues, resulting in the activation of protein tyrosine kinase (PTK). Ultimately, PTK induces the addition of phosphate groups to tyrosine residues in the axoneme of the sperm flagellum, leading to enhanced movement. Another crucial occurrence during capacitation is the activation of calcium ions, which is also initiated by ROS. The rise in calcium ions triggers the splitting of phosphatidylinositol-4,5-biphosphate (PIP2), resulting in the formation of diacylglycerol (DAG). DAG and PKC trigger the phosphorylation of phospholipase A2, an essential membrane enzyme involved in sperm activity. Phosphorylation of the spermatozoa membrane enhances its fluidity, facilitating fusion with the egg. The process of membrane fusion is essential for fertilization and requires accurate synchronization between the sperm and egg. In summary, the interaction among ROS, calcium ions, PKC, and phospholipase A2 during capacitation is a highly regulated mechanism that guarantees effective fertilization (53).

## Conclusion

According to the previous research and the results of this study, it is proposed that engaging in moderate-intensity exercise over a long time can suppress the production of pro-inflammatory cytokines by decreasing oxidative stress. High-Intensity Interval Training (HIIT) may enhance sperm DNA integrity and improve sperm characteristics in males with infertility issues. Oxidative stress is recognized to have detrimental impacts on the male reproductive system, leading to infertility. Because of the elevated rate of cell division and mitochondrial oxygen consumption, as well as the comparatively abundant presence of unsaturated fatty acids, the testicle is more susceptible to oxidative stress (Figure 1).
